# Investigation of Susceptibility Genes Triggering Lachrymal/Salivary Gland Lesion Complications in Japanese Patients with Type 1 Autoimmune Pancreatitis

**DOI:** 10.1371/journal.pone.0127078

**Published:** 2015-05-18

**Authors:** Takaya Oguchi, Masao Ota, Tetsuya Ito, Hideaki Hamano, Norikazu Arakura, Yoshihiko Katsuyama, Akira Meguro, Shigeyuki Kawa

**Affiliations:** 1 Department of Gastroenterology, Shinshu University School of Medicine, Matsumoto, Japan; 2 Department of Legal Medicine, Shinshu University School of Medicine, Matsumoto, Japan; 3 Endoscopic Examination Center, Shinshu University Hospital, Matsumoto, Japan; 4 Department of Pharmacy, Shinshu University Hospital, Matsumoto, Japan; 5 Department of Ophthalmology, Yokohama City University School of Medicine, Yokohama, Kanagawa, Japan; 6 Center for Health, Safety, and Environmental Management, Shinshu University, Matsumoto, Japan; University of Szeged, HUNGARY

## Abstract

Autoimmune pancreatitis (AIP) is a unique form of chronic pancreatitis characterized by high serum IgG4 concentration and a variety of complicating extra-pancreatic lesions. In particular, lachrymal/salivary gland lesions tend to manifest in a highly active AIP disease state, and several genes are speculated to be associated with the onset of this complication. We therefore searched for candidate susceptibility genes related to lachrymal/salivary gland lesions in a genome-wide association study (GWAS) with the GeneChip Human Mapping 500k Array Set (Affymetrix, CA) that was followed by fine mapping of additional single nucleotide polymorphisms (SNPs) in strongly significant genes with TaqMan assays. Venous blood samples were obtained from 50 type 1 AIP patients with lachrymal/salivary gland lesions (A group) and 53 type 1 AIP patients without (B group). The mean values of IgG and IG4 were both significantly different (*P*<0.05) between the groups. SNPs that showed a significant association with the A group at the genome-wide level (*P*<0.0001) were identified and subsequently used in fine SNP mapping of candidate genes. In total, five SNPs had a positive association with complicated AIP (most notably rs2284932 [*P*=0.0000021]) and five SNPs possessed a negative association (particularly rs9371942 [*P*=0.00000039]). Among them, *KLF7*, *FRMD4B*, *LOC101928923*, and *MPPED2* were further examined for complication susceptibility using additional SNPs that were not included in the GWAS. Individual genotyping of *KLF7* rs2284932 revealed that the frequency of the minor C allele was significantly increased (*P*=0.00062, *Pc*=0.0018, OR=2.98, 95%CI=1.58-5.65) in group A. The minor T allele of rs4473559 in *FRMD4* demonstrated a significant association in the A group (*P*=0.00015, OR=3.38, 95%CI=1.77-7.65). In the *LOC101928923* gene, the frequency of the minor C allele of rs4379306 was significantly decreased in group A in both TaqMan and GWAS analyses. Lastly, the minor C allele of *MPPED2* rs514644 carried a significantly increased risk of complications. These four genes may be linked with the onset of lachrymal/salivary gland lesions in type 1 AIP patients and require further study.

## Introduction

Autoimmune pancreatitis (AIP) is a unique form of chronic pancreatitis characterized by the imaging findings of irregular narrowing of the main pancreatic duct, pancreatic swelling, and obstructive jaundice, all of which mimic the clinical signs of pancreatic cancer [1]. AIP patients also exhibit high serum IgG4 concentration, abundant lymphoplasmacytic and IgG4-bearing plasma cell infiltration in pancreatic lesions, and a favorable response to steroid therapy. Although such features indicate that specific autoimmune mechanisms associated with IgG4 are present in AIP [2, 3], its precise pathogenesis has not been fully elucidated.

AIP is complicated with a variety of extra-pancreatic lesions, such as dacryoadenitis/sialadenitis [4, 5], lung lesions [6], sclerosing cholangitis [7, 8], retroperitoneal fibrosis [3], and tubulointerstitial nephritis [9]. Since AIP and these lesions often share the pathological features of prominent IgG4-positive plasma cell infiltration in affected organs and a positive response to corticosteroids, a common pathogenic background is suspected [3]. IgG4-related disease (IgG4-RD) is a newly proposed systemic disorder that encompasses both of these conditions. Accordingly, AIP is now recognized as pancreatic manifestation of IgG4-RD [1].

Dacryoadenitis/sialadenitis occurring in AIP was previously considered to represent Mikulicz disease. However, it is now considered to be a principal member of IgG4-RD and is referred to as IgG4-related dacryoadenitis and sialadenitis [10]. IgG4-related dacryoadenitis and sialadenitis is characterized by symmetrical swelling of the lacrimal and submandibular glands, high serum IgG4 concentration, and abundant IgG4-positive plasma cell infiltration in the affected tissues [4, 11]. Unlike Sjögren’s syndrome, which also exhibits dacryoadenitis and sialadenitis, the IgG4-related variety has no relation to disease-specific autoantibodies, such as anti-SSA or anti-SSB, shows mild or absent exocrine insufficiency, and reacts well to corticosteroid therapy. Moreover, complicating lachrymal/salivary gland lesions tend to manifest in a highly active AIP disease state [11].

Since many autoimmune disorders are associated with multiple genetic and environmental factors, it is generally considered that the development of AIP is influenced by several susceptibility genes, including HLA DRB1*04:05-DQB1*04:*01*, *FCRL3*, *CTLA4*, *KCNA3*, and *TLR4* [12–16]. Among them, HLA class II genes have been genetically characterized as primary predisposition [12, 17] and relapse [18] factors in AIP. However, disease susceptibility remains poorly understood, especially the relationship between relapse and a substitution of aspartic acid at codon 57 of DQ β1 [19]. These, and other, genes are also speculated to be linked to the induction of AIP complicated with dacryoadenitis/sialadenitis and have important clinical significance.

The genome-wide association study (GWAS) method is a powerful and widely-used technique for exploring the relationships among common sequence variations and disease susceptibility or resistance throughout the entire genome. This approach has demonstrated numerous common variants that contribute to disease predisposition and complex traits [20]. To our knowledge, no GWAS has been done on AIP complicated with lachrymal/salivary gland lesions to date. Proper consideration for small sample sizes and sample collection biases is needed to reliably identify disease-susceptible loci using a GWAS [21, 22]. Major autoimmune diseases, such as rheumatoid arthritis, type I diabetes mellitus and systemic lupus erythematosus, have large patient populations from which to sample. Although AIP is a rare disease, we expect that the collection of a well defined cohort using specific clinical diagnostic criteria will enable adequate GWAS analysis. In the present study, we first screened for susceptibility genes of lachrymal/salivary gland lesions in type 1 AIP using the GeneChip Human Mapping 500k Array Set (Affymetrix, CA). Next, fine-tuned mapping of specific single nucleotide polymorphisms (SNPs) was performed for candidate genes that showed a strong statistical significance (*P*<0.0001).

## Materials and Methods

### 1. Patients and Samples

One hundred and nine patients with type 1 AIP (82 men and 26 women, median age at AIP onset: 66 years) were examined and treated at Shinshu University Hospital or its affiliated institutions between August 1992 and August 2012. Among them, we recruited 103 patients who provided consent for inclusion in the GWAS and collected venous blood samples from 50 AIP patients with lachrymal/salivary gland lesions (i.e., the A group) and 53 patients without (i.e., the B group). Collected samples were immediately frozen and stored at minus 80°C until analysis.

The A group consisted of 41 men and 9 women who ranged from 49 to 85 years of age (average: 63.5 years). The B group included 40 men and 13 women who ranged from 38 to 84 years of age (average: 65.1 years). Dacryoadenitis and sialadenitis were defined as symmetrical swelling of the lachrymal and salivary glands as confirmed by physical examination, CT and MRI findings, and gallium scintigraphy.

### 2. Methods

#### 2–1. Comparison of activity state between type 1 AIP with and without lachrymal/salivary gland lesions

To confirm whether type 1 AIP with lachrymal/salivary gland lesions was at a higher disease activity state than AIP without the involvement of lesions, we performed a comparative study between the groups using several activity markers, including IgG, IgG4, circulating immune complex (CIC), β 2-microgloburin (β2MG), soluble interleulkin-2 receptor (sIL2R), and complement C3 and C4, as well as estimation of other organ involvement, such as lung disease, sclerosing cholangitis, kidney disease, or retroperitoneal fibrosis.

### 2–2. Genetic analysis.

#### 2-2-1. Preparation of genomic DNA

Genomic DNA was isolated and purified from venous whole blood samples using a commercially available kit (QuickGene DNA whole blood kit L, Kurabo, Osaka, Japan). All procedures were performed according to the manufacturer’s instructions under standardized conditions to prevent variation in DNA quality.

#### 2-2-2. Genome-wide genotyping

Genotyping with the GeneChip Human Mapping 500K Array Set was carried out according to the manufacturer’s protocol for our first stage of analysis. Samples with a <93% genotype call rate were excluded from the study, as were SNPs with a call rate of <95% or a minor allele frequency of <5% overall.

#### 2-2-3. SNP genotyping

To specifically identify possible susceptibility genes of AIP-complicating dacryoadenitis and sialadenitis, SNPs that showed a strongly significant association in the A group at the genome-wide level (*P*<0.0001) were assessed. Among them, we selected four candidate genes (Kruppel-like factor 7 [*KLF7*], FERM domain containing 4B [*FRMD4B*], uncharacterized LOC1928923 [*LOC101928923*], and metallophosphor esterase domain-containing protein [*MPPED2*]) (Table 1) and examined tagging SNPs in these genes as the second stage of our analysis. The selection criteria for the SNPs were based on information from the NCBI dbSNP database (build 37.3, http://www.ncbi.nlm.nih.gov/projects/SNP/), HapMap database (http://hapmap.ncbi.nlm.nih.gov/downloads/index.html.en), and SNP database of Applied Biosystems (http://bioinfo.appliedbiosystems.com/genome-database/snp-genotyping.html) as: 1) location within the candidate gene; 2) minor allele frequency >5% in Japanese populations; 3) call rate ≧95%; and 4) Hardy-Weinberg equilibrium *P*≧0.001. Genotyping of all SNPs was performed using the ABI TaqMan allelic discrimination kit and the ABI7500 Sequence Detection System (Applied Biosystems, Carlsbad, CA) following the manufacturer’s instructions.

**Table 1:** Single nucleotide polymorphisms showing the strongest associations (P<0.0001) in the genome-wide association study.

dbSNP ID	Chrom. location	Position	Candidate gene	MA	*P* value	OR (95% CI)
rs2284932	2q33.3	207720754	KLF7	C	<0.000003	4.35 (2.32–8.16)
rs9831516	3p14.1	69312751	FRMD4B	G	<0.00002	3.11 (1.72–5.62)
rs2407212	5q23.2	121912400	SNCAIP	G	<0.00009	6.20 (2.28–16.91)
rs524762	6q13	75112962	COL12A1	T	<0.00003	0.31 (0.17–0.53)
rs9371942	6q25.3	156276214	LOC101928923	G	<0.0000004	0.20 (0.10–0.42)
rs4735508	8q22.1	98986987	MATN2	A	<0.0001	8.42 (2.43–29.14)
rs1536067	9p22.2	17727893	SH3GL2	G	<0.00004	0.24 (0.12–0.48)
rs4878053	9q21.33	89102948	FLJ45537	T	<0.00001	0.29 (0.17–0.51)
rs514644	11p14.1	30408757	MPPED2	C	<0.00006	3.06 (1.77–5.30)
rs7170215	15q25.3	86299145	NTRK3	A	<0.00009	0.27 (0.13–0.53)

dbSNP ID: SNP database identification, Chrom: chromosome, Position: distance from the short-arm telomere, MA: minor allele, OR: odds ratio, CI: confidence interval.

#### 2–3. Statistical analysis

Fisher’s exact and Pearson’s chi-square tests were adopted to test for differences in clinical data between the patient subgroups. The Mann-Whitney *U* test was employed to compare continuous data. All tests were performed using Statflex ver. 6 (Artech Co., Ltd., Japan). *P* values of less than 0.05 were considered to be statistically significant.

All association analyses between group A and group B for GWAS data were carried out using HelixTree SVS 7 software (Golden Helix, Inc., Bozeman, MT). The statistical significances of allele frequencies between AIP with and without lachrymal/salivary gland lesions in second stage analyses were calculated using the chi-square test. A *P* value of less than 0.05 was considered to be statistically significant after adjustment by Bonferroni’s correction. The Hardy-Weinberg equilibrium of all SNPs was confirmed.

### 3. Ethics statement

The present study was approved by the Ethics Committee of Shinshu University School of Medicine (Matsumoto, Japan). The protocol of this investigation was in accordance with the principals outlined in the Declaration on Helsinki of the World Medical Association and was approved by the Ethics Committee of Shinshu University School of Medicine. Written informed consent was obtained from each subject after a full explanation of the study.

## Results

### 1. Comparison of activity state in type 1 AIP with and without lachrymal/salivary gland lesions

Serum IgG and IgG4 concentrations were significantly higher in the A group than in the B group. No remarkable associations were observed for CIC, β2MG, sIL2R, C3, or C4 between the groups. Significantly higher prevalences of kidney disease and retroperitoneal fibrosis were detected in the A group. Taken together, type 1 AIP with lachrymal/salivary gland lesions appeared to be in a more highly activated state (Table 2).

**Table 2:** Comparison of activity state between type 1 AIP with and without lachrymal/salivary gland lesions.

	A group	B group	*P* value
	median (range)	median (range)	
IgG	2437.5 (1199–6408)	1865 (892–4661)1865(892–4661)1865(892–4661)	0.00035
IgG4	773 (33–2970)	379 (4–1950)	0.00062
CIC	6.20 (2–41.6)	5 (1.4–58.4)	0.42
β2MG	2.315 (1.3–8.9)	2.165 (1.2–15.3)	0.23
sIL2R	869 (345–4695)869(345–4695)	755 (257–2260)	0.10
C3	102.5 (16–218)	104.5 (12–238)	0.74
C4	21.8 (1.1–152)	23.3 (1.0–162)	0.74
lung disease (+/-)	18/31	10/40	0.10
Sclerosing cholangitis (+/-)	5/42	7/44	0.64
Kidney disease (+/-)	15/35	4/48	0.0038
Retroperitoneal fibrosis (+/-)	18/32	9/42	0.043

A group: with lachrymal/salivary gland lesions, B group: without lachrymal/salivary gland lesions.

### 2. Genetic analysis

We preliminarily conducted a GWAS screening analysis of Japanese type 1 AIP patients with and without lachrymal/salivary gland lesions. Of the total of 12,033 SNPs that passed the internal quality control, 242 exhibited a statistical significance (*P*<0.001) in allele-based tests. The results for strong signals (*P*<0.0001) are shown in Table 2. Five SNPs demonstrated a positive association with complicated AIP: rs2284932 (OR = 4.35), rs9831516 (OR = 3.11), rs2407212 (OR = 6.20), rs4735508 (OR = 8.42), and rs514644 (OR = 3.06). Five SNPs were negatively associated with complicated AIP: rs524762 (OR = 0.31), rs9371942 (OR = 0.20), rs1536067 (OR = 0.24), rs4878053 (OR = 0.29), and rs7170215 (OR = 0.27). The strongest associations with the pathogenesis of lachrymal/salivary gland lesions were rs9371942 (*P* = 0.00000039) and rs2284932 (*P* = 0.0000021).

The candidate genes containing the highly significant SNPs are listed in Table 1 according to data from the NCBI Map Viewer (http://www.ncbi.nlm.nih.gov/mapview/). Among the 10 genes, *KLF7* (2q33.3), *FRMD4B* (3p14.1), *LOC101928923* (6q25.3), and *MPPED2* (11p14.1) were further examined for conferring susceptibility to complications using SNPs that resided in the genes but were not tested in the GWAS. Individual genotyping of *KLF7* rs2284932 using a TaqMan assay showed that the frequency of the minor C allele was significantly increased (*P* = 0.00062, OR = 2.98) in the A group as in the GWAS (Table 3).

**Table 3:** Association analysis of single nucleotide polymorphisms in the KLF7 gene.

dbSNP ID	Chrom. location	Typing method	Alleles	Frequency (%)			*P* value		*Pc* value	OR (95% CI)
					A group	B group				
rs2287505	207655331	GWAS	C>A				A	0.027		2.74(1.09–6.90)
rs1263615	207667483	TaqMan	A>G	A	74.0	68.6	G	0.758	3.032	1.10(0.61–1.96)
				G	26.0	31.4	GG+GA/AA	0.567	2.268	1.26(0.57–2.81)
rs768090	207711824	TaqMan	A>T	A	74.0	83.3	T	0.091	0.364	1.80(0.91–3.57)
				T	26.0	16.7	TT+TA/AA	0.047	0.188	2.28(1.01–5.16)
rs10195536	207715065	GWAS	T>A				A	0.016		3.79(1.21–11.92)
rs2284932	207720754	TaqMan	T>C	T	90.0	95.1	C	0.00062	0.003	2.98(1.58–5.65)
				C	10.0	4.9	CC+AC/AA	0.00037	0.002	4.37(1.90–10.02)
		GWAS					C	0.0000021		4.35(2.32–8.16)
rs12466923	207721800	TaqMan	A>C	A	60.0	81.4	C	0.039	0.156	2.16 (1.03–4.54)
				C	40.0	18.6	CC+AC/AA	0.0093	0.037	3.10(1.30–7.38)

A group: with lachrymal/salivary gland lesions, B group: without lachrymal/salivary gland lesions, dbSNP ID: SNP database identification, Chrom: chromosome, Pc: corrected P.

The minor T allele of rs4473559 in *FRMD4* also had a significant association in the A group (*P* = 0.00015, OR = 3.38) (Table 4).

**Table 4:** Association analysis of single nucleotide polymorphisms in the FRMD4 gene.

dbSNP ID	Chrom. location	Typing method	Alleles	Frequency (%)			*P* value		*Pc* value	OR (95% CI)
					A group	B group				
rs12637416	69302498	GWAS	T>A				A	0.00062		2.73(1.52–4.89)
rs6763046	69302897	TaqMan	C>A	A	40.0	18.6	A	0.00084	0.0034	2.91(1.54–5.52)
				C	60.0	81.4	AA+AC/CC	0.00050	0.002	4.25(1.85–9.76)
		GWAS					A	0.00035		2.91(1.60–5.29)
rs4473559	69305553	TaqMan	G>T	T	42.0	17.6	T	0.00015	0.0006	3.38(1.77–6.45)
				G	58.0	82.4	TT+TG/GG	0.000045	0.00018	5.62(2.39–13.21)
rs4464459	69306951	TaqMan	C>A	A	36.0	16.7	A	0.0018	0.0072	2.81(1.45–5.45)
				C	64.0	83.3	AA+AC/CC	0.00048	0.0019	4.31(1.86–9.98)
rs11128118	69308278	GWAS	G>T				T	0.00019		3.02(1.67–5.45)
rs9831516	69312751	GWAS	A>G				G	0.00013		3.11(1.72–5.62)
rs9836305	69313491	TaqMan	A>G	G	42	18.6	A	0.00030	0.0012	3.16(1.67–5.98)
				A	58	81.4	AA+AG/GG	0.00050	0.002	4.25(1.85–9.76)

A group: with lachrymal/salivary gland lesions, B group: without lachrymal/salivary gland lesions, dbSNP ID: SNP database identification, Chrom: chromosome, Pc: corrected P.

In the *LOC101928923* gene, the frequency of the minor C allele of rs4379306 was significantly decreased (*P* = 0.00017, OR = 0.30) in both TaqMan and GWAS analyses in the A group (Table 5).

**Table 5:** Association analysis of single nucleotide polymorphisms in the LOC101928923 gene.

dbSNP ID	Chrom. location	Typing method	Alleles	Frequency (%)			*P* value		*Pc* value	OR (95% CI)
					A group	B group				
rs9371942	156276214	GWAS	A>G				G	0.0000039		0.20(0.10–0.42)
rs4379306	156276633	GWAS	A>T				T	0.0000089		0.21(0.10–0.44)
		TaqMan		T	17.0	32.4	T	0.011	0.033	0.43(0.22–0.83)
				A	83.0	67.6	TT+TA/AA	0.0065	0.020	0.33(0.14–0.74)
rs9397861	156279496	TaqMan	A>G	G	16.0	36.3	G	0.0011	0.0033	0.33(0.17–0.65)
				A	84.0	63.7	GG+GA/AA	0.0019	0.0057	0.28(0.12–0.63)
rs4428513	156288830	TaqMan	T>C	C	8.0	7.8	C	0.97		1.02(0.37–2.84)
				T	92.0	92.2	CC+CT/TT	0.75		1.20(0.40–3.59)
rs9371408	156307788	GWAS	A>G				G	0.000029		0.22(0.11–0.47)
rs9384400	156323446	GWAS	A>T				T	0.000014		0.22(0.11–0.45)

A group: with lachrymal/salivary gland lesions, B group: without lachrymal/salivary gland lesions, dbSNP ID: SNP database identification, Chrom: chromosome, Pc: corrected P.

Lastly, the minor C allele for rs514644 of *MPPED2* carried a significantly increased risk for complicating lachrymal/salivary gland lesions (*P* = 0.0075, OR = 2.14) (Table 6).

**Table 6:** Association analysis of single nucleotide polymorphisms in the MPPED2 gene.

dbSNP ID	Chrom. location	Typing method	Alleles	Frequency (%)			*P* value		*Pc* value	OR (95% CI)
					A group	B group				
rs10835665	30406319	TaqMan	G>A	A	26.0	19.6	A	0.28		1.44(0.74–2.79)
				G	74.0	80.4	AA+AG/GG	0.49		1.33(0.59–2.96)
rs514644	30408757	TaqMan	T>C	C	59.0	40.2	C	0.0075	0.045	2.14(1.22–3.75)
				T	41.0	59.8	CC+CT/TT	0.017	0.102	2.63(1.01–6.82)
		GWAS					C	0.000054		3.06(1.77–5.30)
rs487742	30410971	TaqMan	G>A	A	66.0	45.1	A	0.0028	0.017	2.36(1.34–4.17)
				G	34.0	54.9	AA+AG/GG	0.051	0.306	2.77(0.97–7.94)
rs808182	30411818	TaqMan	G>A	A	33.0	20.6	A	0.046	0.276	1.90(1.01–3.59)
				G	67.0	79.4	AA+AG/GG	0.036	0.216	2.35(1.05–5.25)
rs11031087	30415467	TaqMan	A>T	T	7.0	4.9	T	0.53		1.46(0.45–4.76)
				A	93.0	95.1	TT+AT/AA	0.51		1.50(0.44–5.08)
rs11031093	30424076	TaqMan	G>A	A	7.0	4.9	A	0.53		1.46(0.45–4.76)
				G	93.0	95.1	AA+AG/GG	0.51		1.50(0.44–5.08)
rs537944	30434891	GWAS	G>A				A	0.000052		3.07(1.77–5.32)
rs521436	30449780	GWAS	T>A				A	0.000099		2.92(1.69–5.04)

A group: with lachrymal/salivary gland lesions, B group: without lachrymal/salivary gland lesions, dbSNP ID: SNP database identification, Chrom: chromosome, Pc: corrected P.

## Discussion

AIP is believed to be a pancreatic manifestation of a systematic IgG4 disorder that is subclassified as either IgG4-related (type 1) or non-IgG4-related (type 2) [1]. Type 1 AIP tends to exhibit lesions in various other organs, including the lachrymal and salivary glands, lungs, retroperitoneum, and prostate.

We observed in the present study that approximately 50% of patients had lachrymal/salivary lesion involvement despite other reports showing the prevalence of these lesions to be approximately 20% in Japan [23, 24]. Since lachrymal/salivary lesions are considered to be a major member of the IgG4-RD family along with Mikulicz’s disease, their incidence in AIP is presumably high. Although the reason for such a discrepancy in positivity rates for these lesions is not precisely clear, it may be attributable to differences in diagnostic procedures, such as physical examinations or imaging tests. In our previous study, imaging analysis of AIP by an experienced radiologist disclosed the presence of extra-pancreatic lesions in 92% of AIP patients and lachrymal/salivary lesions in 47.5% of cases [25].

Although type 1 AIP complicated with lachrymal/salivary gland lesions can be clearly diagnosed using recent clinical, immunological, radiological, and morphological characterization criteria [11], little is known on the pathogenesis of these complications. Therefore, we investigated whether genetic factors affected the development of lachrymal/salivary gland lesions in type 1 AIP using a GWAS followed by fine mapping of additional SNPs and uncovered four novel candidate susceptibility genes.

The initial GWAS revealed 10 candidate genes possibly influencing the pathogenesis of lachrymal/salivary gland lesions in AIP. As several genes contained multiple SNPs that were strongly associated with complications, we selected four (positive association: *KLF7*, *FRMD4*, and *MPPED2*; negative association: *LOC101928923*) according to *P* values and OR for ensuing minor allele analysis.

The *KLF7* gene encodes a member of the Kruppel-like factors among DNA-binding transcriptional regulators that play diverse roles during cell proliferation and differentiation [26–28]. KLF7 is reportedly related to neurogenesis [29], progression of type 2 diabetes [30], obesity [31], and regulation of thymocyte development [32]. Determining the specific role of *KLF7* polymorphisms in the onset of lachrymal/salivary gland lesions appears challenging at present. However, they may contribute to disease complications as shown in the individual genotyping results in Table 3 (C allele at rs2284932 [*P* = 0.00037] with a dominant model and C allele at rs12466923 [*P* = 0.0093] with a dominant model).

The *FRMD4B* gene is ubiquitously expressed and encodes a GRP1-binding protein (GRSP1) that contains a FERM protein domain [33]. FRMD4B might be involved in the establishment of epithelial cell polarity and play a role as a scaffolding molecule [34]. This protein also participates in activated insulin receptor signaling complexes and performs functions in insulin receptor, growth factor receptor, and other phosphatidylinoshitol (3,4,5)-trisphosphate (PIP3) signaling events [35]. To date, few disease associations have been made with *FRMD4B* polymorphisms. Our study showed that all SNPs had strongly significant associations (*P*<0.001) with lachrymal/salivary gland lesions in both TaqMan and GWAS typing. The precise involvement of *FRMD4B* remains unknown, but we speculate that polymorphisms may affect complication onset based on previous functional information [35].

All statistically associated SNPs (rs9371942, rs4379306, and rs9397861) were located in the *LOC101928923* gene on chromosome 6p25.3. This little known gene resides in the short intergenic region between *NOX3* and *MIR1202*. NOX3 is a member of the NOX family of NADPH oxidases. NOX enzymes are a potential source of reactive oxygen species (ROS) production that transport electrons across the plasma membrane [36]. NOX3-derived ROS appear to be associated with numerous biological functions, including insulin action, host defense, cellular signaling, regulation of gene expression, and cell differentiation [37]. *NOX3* polymorphisms may be in high linkage disequilibrium with the three candidate SNPs uncovered in *LOC101928923* and influence the induction of complications. Further association studies using SNPs in *NOX3* might determine whether *NOX3* polymorphisms affect the pathogenesis of complications.

The frequencies of two minor alleles (C at rs514644 and A at rs487742) in *MPPED2* were significantly increased in the A group over the B group (*P* = 0.0075 and *P* = 0.0028, respectively). The *MPPED2* gene (also known as *c11orf8* or *239FB*) is located on human chromosome 11p13 between the *FSHB* and *PAX6* genes. The upregulation of *MPPED2* reduces cell proliferation, induces apoptosis, and stimulates the differentiation of neuronal precursors [38]. Particularly in papillary thyroid carcinomas and breast cancer, MPPED2 expression has been reported to affect the malignancy of lesions [39–41]. Therefore, *MPPED2*-regulated anti-tumorigenesis may play an important role in the induction or regulation of lachrymal/salivary gland lesions.

The number of enrolled subjects for this rare disease was too small to overcome type I statistical error. However, the false positive report probability (FPRP) values as calculated by Wacholder’s method (http://jnci.oxfordjournals.org/content/96/6/434/suppl/DC1) [42] support the significant findings revealed in the present study, i.e., when a prior probability of 0.05 was set, FPRP values were 0.018 (dominant model) with rs2284932 in *KLF7*, 0.003 (dominant model) with rs4473559 in *FRMF4*, 0.0049 (additive model) with rs9397861 in *LOC101928923*, and 0.106 (additive model) with rs487742 in *MPPED2*.

In conclusion, we identified four novel candidate genes (*KLF7*, *FRMD4B*, *NOX3*, and *MPPED2*) that might be linked to the development of lachrymal/salivary gland lesions in type 1 AIP patients using a GWAS followed by fine mapping of highly significant genes. Further studies using larger sample sizes and functional analysis of genes associated with AIP complications are needed to confirm the present results.
