# Correction: Investigation of Susceptibility Genes Triggering Lachrymal/Salivary Gland Lesion Complications in Japanese Patients with Type 1 Autoimmune Pancreatitis

**DOI:** 10.1371/journal.pone.0146738

**Published:** 2016-01-05

**Authors:** Takaya Oguchi, Masao Ota, Tetsuya Ito, Hideaki Hamano, Norikazu Arakura, Yoshihiko Katsuyama, Akira Meguro, Shigeyuki Kawa

There are errors in the ninth, tenth and eleventh sentences of the Abstract. The correct sentences are: Individual genotyping of *KLF7* rs2284932 revealed that the frequency of the minor C allele was significantly increased (*P* = 0.00062, *Pc* = 0.003, OR = 2.98, 95%CI = 1.58–5.65) in group A. The minor T allele of rs4473559 in *FRMD4* demonstrated a significant association in the A group (*P* = 0.00015, OR = 3.38, 95%CI = 1.77–6.45). In the *LOC101928923* gene, the frequency of the minor T allele of rs4379306 was significantly decreased in group A in both TaqMan and GWAS analyses. Lastly, the minor C allele of *MPPED2* rs514644 carried a significantly increased risk of complications.

There is an error in the third sentence of the Genetic Analysis section of the Results. The correct sentence is: The results for strong signals (*P*<0.0001) are shown in Table 1. There are also errors in the penultimate sentence of this section. The correct sentence is: In the *LOC101928923* gene, the frequency of the minor T allele of rs4379306 was significantly decreased (*P* = 0.011, OR = 0.43) in both TaqMan and GWAS analyses in the A group (Table 5).

There is an error in Table 3. The *P* value for rs2284932 should be CC+TC/TT. Please see the corrected Table 3 here.

**Table 3:** Association analysis of single nucleotide polymorphisms in the KLF7 gene.

dbSNP ID	Chrom. location	Typing method	Alleles	Frequency (%)			*P* value		*Pc* value	OR (95% CI)
					A group	B group				
rs2287505	207655331	GWAS	C>A				A	0.027		2.74(1.09–6.90)
rs1263615	207667483	TaqMan	A>G	A	74.0	68.6	G	0.758	3.032	1.10(0.61–1.96)
				G	26.0	31.4	GG+GA/AA	0.567	2.268	1.26(0.57–2.81)
rs768090	207711824	TaqMan	A>T	A	74.0	83.3	T	0.091	0.364	1.80(0.91–3.57)
				T	26.0	16.7	TT+TA/AA	0.047	0.188	2.28(1.01–5.16)
rs10195536	207715065	GWAS	T>A				A	0.016		3.79(1.21–11.92)
rs2284932	207720754	TaqMan	T>C	T	90.0	95.1	C	0.00062	0.003	2.98(1.58–5.65)
				C	10.0	4.9	CC+TC/TT	0.00037	0.002	4.37(1.90–10.02)
		GWAS				C	0.0000021		4.35(2.32–8.16)
rs12466923	207721800	TaqMan	A>C	A	60.0	81.4	C	0.039	0.156	2.16 (1.03–4.54)
				C	40.0	18.6	CC+AC/AA	0.0093	0.037	3.10(1.30–7.38)

A group: with lachrymal/salivary gland lesions, B group: without lachrymal/salivary gland lesions, dbSNP ID: SNP database identification, Chrom: chromosome, Pc: corrected P
